# New Carvone-Based Deep Eutectic Solvents for Siloxanes Capture from Biogas

**DOI:** 10.3390/ijms22179551

**Published:** 2021-09-02

**Authors:** Patrycja Makoś-Chełstowska, Edyta Słupek, Aleksandra Kramarz, Jacek Gębicki

**Affiliations:** 1 Department of Process Engineering and Chemical Technology, Faculty of Chemistry, Gdansk University of Technology, 80-233 Gdansk, Poland; patrycja.makos@pg.edu.pl (P.M.-C.); edyta.slupek@pg.edu.pl (E.S.); olakramarz96@gmail.com (A.K.); 2 EcoTech Center, Gdańsk University of Technology, 80-233 Gdańsk, Poland

**Keywords:** biogas, deep eutectic solvents, siloxanes, conductor-like screening model for real solvents

## Abstract

During biogas combustion, siloxanes form deposits of SiO_2_ on engine components, thus shortening the lifespan of the installation. Therefore, the development of new methods for the purification of biogas is receiving increasing attention. One of the most effective methods is physical absorption with the use of appropriate solvents. According to the principles of green engineering, solvents should be biodegradable, non-toxic, and have a high absorption capacity. Deep eutectic solvents (DES) possess such characteristics. In the literature, due to the very large number of DES combinations, conductor-like screening models for real solvents (COSMO-RS), based on the comparison of siloxane activity coefficient of 90 DESs of various types, were studied. DESs, which have the highest affinity to siloxanes, were synthesized. The most important physicochemical properties of DESs were carefully studied. In order to explain of the mechanism of DES formation, and the interaction between DES and siloxanes, the theoretical studies based on σ-profiles, and experimental studies including the ^1^H NMR, ^13^C NMR, and FT-IR spectra, were applied. The obtained results indicated that the new DESs, which were composed of carvone and carboxylic acids, were characterized by the highest affinity to siloxanes. It was shown that the hydrogen bonds between the active ketone group (=O) and the carboxyl group (-COOH) determined the formation of stable DESs with a melting point much lower than those of the individual components. On the other hand, non-bonded interactions mainly determined the effective capture of siloxanes with DES.

## 1. Introduction

Currently, increasing attention is being paid to obtaining so-called “green energy” from waste materials [1,2,3,4]. An alternative to natural gas is biogas, which is currently produced in over 17,783 biogas plants located in Europe. However, not all biogas plants have the technology needed for effective biogas purification that is in line with the parameters of high-methane gas. According to data from 2020 in Europe, only 729 biogas plants produced high-methane gas. In Europe, the leader in the production of biomethane is Germany, which currently has 232 biogas plants equipped with a system for producing high-quality methane. France and the United Kingdom have 131 and 80 such technologies, respectively [5]. In Poland, there are over 300 biogas plants, and only a few of them are capable of producing biomethane [6]. Biogas that is not fully purified cannot be injected into the transmission grid or used as an alternative transport fuel [7].

Biogas is a product of the methane fermentation process, in which the raw materials are mostly agricultural, landfill, and sewage waste. Raw biogas has a very rich chemical composition. The composition of biogas is strongly dependent on the raw material used for its production. Biogas mainly consists of methane (CH_4_) and carbon dioxide (CO_2_). In addition, biogas contains a lot of impurities including inorganic gaseous compounds, i.e., nitrogen, oxygen, hydrogen, ammonia, carbon monoxide and water vapor, and numerous volatile organic compounds (VOC), including sulfur, nitrogen, oxygen-containing compounds, siloxanes, terpenes, linear and aromatic hydrocarbons [8,9,10]. The total concentration of organic and inorganic impurities may even amount to 65% of the volume of the obtained biogas, constituting a ballast that significantly reduces its calorific value. The calorific value of pure CH_4_ is 35.7 MJ/m^3^, while the calorific value of biogas ranges from 16 to 23 MJ/m^3^ [6]. In addition, biogas impurities can be harmful in many biogas applications, and they can have environmental impacts such as stratospheric ozone depletion, increasing of the greenhouse effect, and reductions in local air quality [11,12,13]. One of the more troublesome groups of pollutants is organosilicon compounds (siloxanes). During biogas combustion, trace amounts of siloxanes are converted into SiO_2_ deposits and damage the engine surfaces. Therefore, biogas needs to be upgraded before combustion [14].

Treatment biogas methods can be classified into adsorption, physical and chemical absorption, biological, cryogenic, and membrane separation methods [15,16,17,18,19]. These methods are mainly used for the removal of carbon dioxide, water, ammonia, and hydrogen sulfide, and they are not suitable for removing volatile organic compounds from biogas [20,21]. Absorption is one of the oldest and best-known technologies for gas purification. Until now, absorption has been mainly used for the removal of inorganic chemical compounds such as carbon dioxide, hydrogen sulfide, siloxanes, and other trace contaminants from gaseous fuel streams. Most absorption processes use organic solvents as absorbents. Physical scrubbing based on organic solvents has many advantages over the main technologies used for biogas treatment. These include:

- Very high treatment efficiency with a properly selected solvent;
- Low methane losses (below 1%);
- Simple and relatively inexpensive technology;
- The capacity to be regenerated many times.

However, most of the used absorbents are substances that negatively affect the natural environment and require large amounts of energy for regeneration [22,23]. Due to this, it is necessary to explore new green biogas purification methods which will be effective for the removal of hydrophobic VOCs including siloxanes.

Data from the literature suggest ionic liquids (ILs) as promising media for VOCs capture from biogas, due to their unique properties [24,25] However, in recent years, it was shown that ILs can be toxic and non-biodegradable, and that their synthesis can be very costly and complicated [26]. Despite the high efficiency of VOCs removal, the disadvantages of ILs preclude their use in real industrial processes. In the last few years, new absorption media called deep eutectic solvents (DESs) were studied in detail. DES is a mixture of two or more chemical components, one of which is a hydrogen bond acceptor (HBA), and the second is a donor (HBD). Due to the formation of strong hydrogen bonds between components, DESs are characterized by significant depressions in melting points (MP) compared to pure compounds. DESs have similar physicochemical properties to ILs, i.e., negligible vapor pressure, non-volatility, thermal stability, high conductivity, and tunable miscibility. Additionally, they are also non-toxic and biodegradable, and their synthesis is simple and cheap [27,28,29]. Due to the favorable properties of DESs, they are used in many processes, i.e., extraction [30,31,32,33,34], sample preparation [35], absorption [36,37,38,39], adsorption [40], metal electrodeposition [41], and biotransformations [42]. In addition, there are a lot of studies on the application of DESs for the removal of inorganic contaminants from gas streams, i.e., those of CO_2_ [43,44,45], H_2_S [46], SO_2_ [47], H_2_O [48], NH_3_ [49]. However, there is very little work on the capture of siloxanes using DESs. 

The paper presents a screening of 90 new non-ionic hydrophobic deep eutectic solvents composed of natural ingredients, i.e., terpenes, carboxylic acids, and polyphenols. For this purpose, the conductor-like screening model for real solvents (COSMO-RS) based on the comparison of siloxane activity coefficient to various DESs was used. Due to the fact that new never-before-published carvone mixtures with organic acids were selected as the most effective DES absorbents, the detailed results of structural characterization of new DESs, and their basic physicochemical properties, i.e., melting point, density, and viscosity, were described. The mechanism of siloxane absorption by DESs was studied by means of ^1^H NMR, ^13^C NMR and FT-IR spectroscopy, based on σ-profiles. In addition, the optimization studies of the main parameters affecting the siloxane absorption process were considered. The possibility of the DESs’ regeneration was also examined. 

## 2. Results and Discussion

### 2.1. Screening of DES 

**Figure 1:**
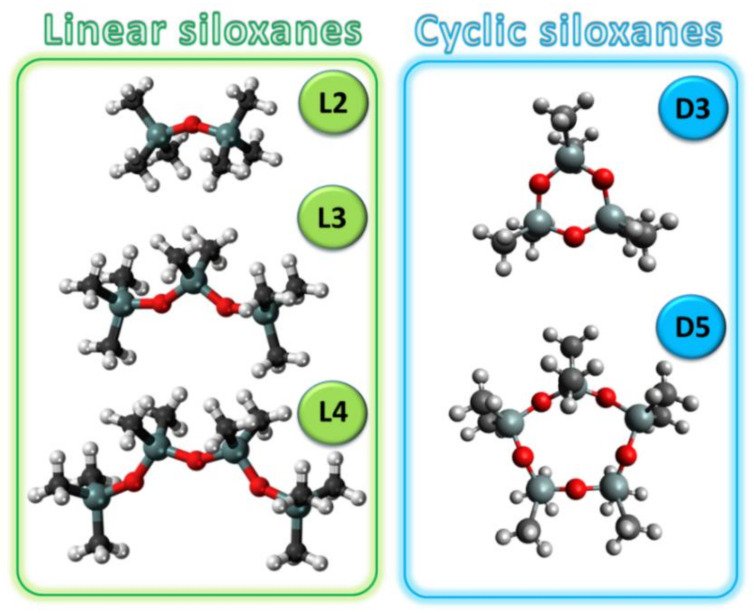
Structures of siloxanes used in the studies.

In the studies, the COSMO-RS model was used for the screening of DESs, which were characterized by the highest affinity to siloxanes. The screening tests were prepared for the most common siloxanes in biogas, i.e., hexamethyldisiloxane (L2), octamethyltrisiloxane (L3), decamethyltetrasiloxane (L4), hexamethylcyclotrisiloxane (D3), and decamethylcyclopentasiloxane (D5), which represented linear and cyclic siloxanes [13,50,51] The structures of siloxanes are presented in Figure 1.

**Figure 2:**
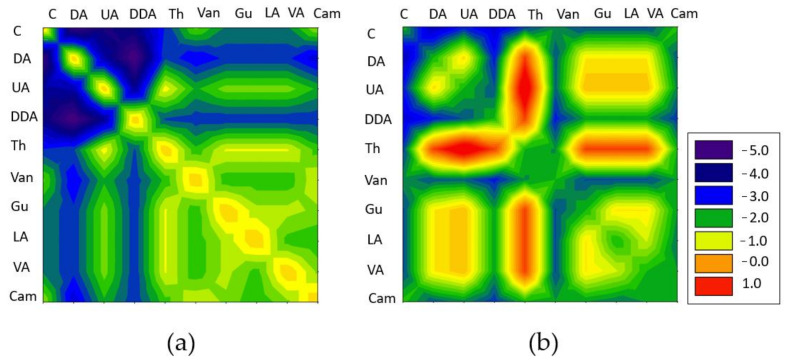
Screening of logarithmic activity coefficients of (**a**) L2 and (**b**) D3 in various DES mixtures at a 1:1 molar ratio.

As solvents, DESs composed of two chemical components, which could be easily extracted from natural sources, i.e., plants or biomass, were tested. All components, i.e., carvone (C), camphor (Cam), thymol (Th), vanillin (V), guaiacol (Gu), levulinic acid (LA), vanillin acid (VA), decanoic acid (DA), undecanoic acid (UA), and dodecanoic acid (DDA), were mixed with each other at a 1:1 molar ratio. Based on the previous studies, the logarithmic activity coefficient (ln (1/γ)) was used to select the DES that exhibited the highest solubility of the siloxanes. This is a parameter that is directly related to the strength of interactions between molecules. The more negative the value, the greater the strength of the dominant interactions between siloxanes and DES, which indicates a greater affinity of solutes to solvents. In turn, the greater affinity of siloxanes to DES is responsible for their greater solubility. All calculations were performed at 20 °C and 101.325 kPa. The calculation results are presented in Figure 2. The paper presents only the most representative results for L2 and D3, which illustrated linear and cyclic siloxanes. The graphical results for the remaining siloxanes were omitted, as they fully corresponded to those obtained for L2 and D3. The obtained results indicated that L2 had a high affinity to most of the tested DES. However, completely different results were obtained for cyclic siloxane-D3. For this compound, lower logarithmic activity coefficients (ln (1/γ) values) were obtained for DESs that were composed of carvone and carboxylic acids, i.e., decanoic, undecanoic acid, and dodecanoic acid, at a 1:1 molar ratio. The reason for the greater solubility of siloxanes in DESs that were composed of carvone and carboxylic acids was probably their specific simple structures. The carvone had a carbonyl group that was capable of forming hydrogen bonds with the carboxyl group of DA, UA and DDA. The lack of additional groups in the HBA and HBD structures meant that oxygen from the Si−O−Si group in siloxanes had better access to the carboxyl group to which it could attach. The presence of other groups in the remaining DES, composed of, i.e., = O, -O-CH_3_, -CH_3_, caused the active sites to become covered, and thus, the interaction strength between the siloxanes and DES was reduced. 

**Figure 3:**
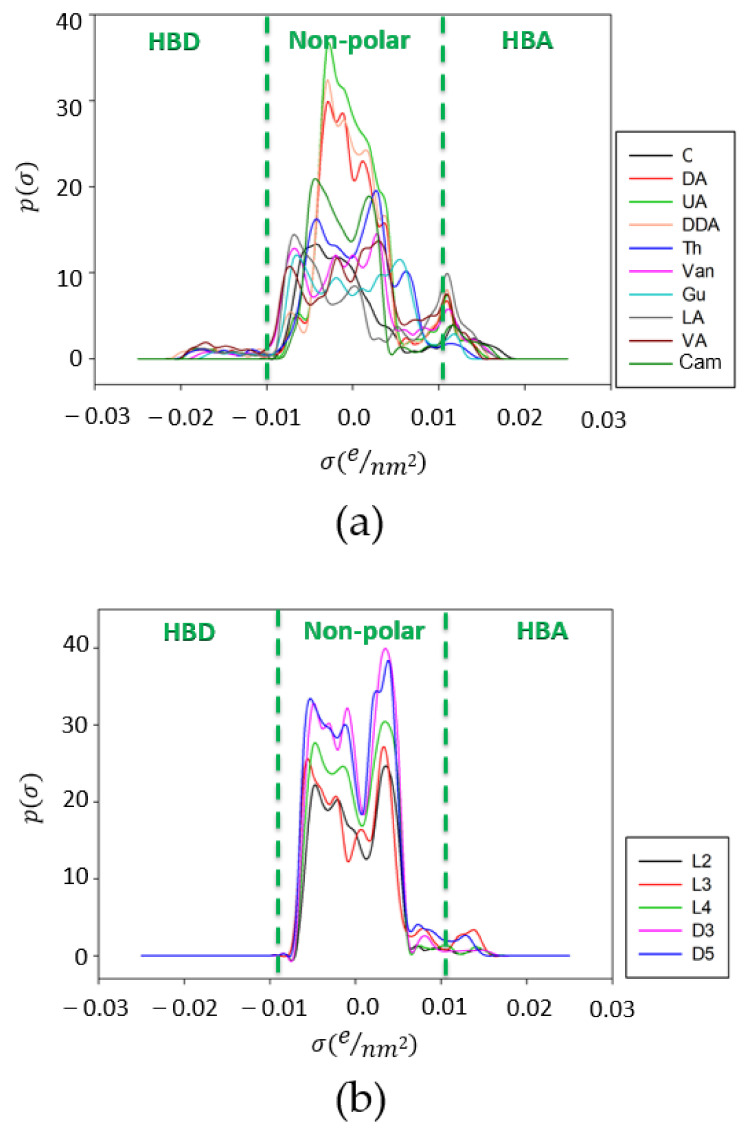
σ-profiles of (**a**) DES components and (**b**) siloxanes.

In order to explain the obtained results, the σ-profiles of DESs’ components and selected siloxanes were analyzed. The σ-profile is the molecule-specific property that determines the probability distribution of the surface area of molecules that have charge density [52,53]. Diagrams with σ-profiles of all studied molecules are presented in Figure 3. The σ-profile diagram could be divided into three segments, including an HBA region in the range of −0.025 eÅ^−2^ < σ < 0.01 eÅ^−2^, an HBD region in the range of 0.01 eÅ^−2^ < σ < 0.025 eÅ^−2^, and the segment between HBA and HBD that represents the non-polar region. Both the hydrogen bond donor and hydrogen bond acceptor segments indicated the potential of the studied components to form strong H bonds. The results for the σ-profiles indicated that DESs’ components, depending on their structure, could be classified as hydrogen bond acceptor or donor groups. The first group was the HBA components, among which Cam and C could be distinguished, due to the active ketone group (=O) in the structures. The second group was that of the HBD components, to which Thy belonged due to the presence of a hydroxyl group (–OH) in the structure. Theoretically, all fatty acids (DA, UA, and DDA) could also be adapted to the HBD group. However, due to the specific structure of the carboxylic group (-COOH), fatty acids could be both donors and acceptors of hydrogen bonds. Therefore, they could be included in the third group, which also included Van (-CHO; -OH; -O-CH_3_), Gu (-OH; -O-CH_3_), LA (-COOH; = O) and VA (-COOH; -O-CH_3_; -OH). These components could theoretically be mixed with each other and form hydrogen bonds, which could determine the formation of a stable eutectic mixture. For all the studied DES components, and siloxanes, a large peak in the non-polar region could be observed. This indicated that the siloxanes had a mostly non-polar character. Only small peaks around the HBA region could be observed in the σ-profile diagram of siloxanes, which indicated that siloxanes could also form H bonds with the –OH or –COOH group.

**Figure 4:**
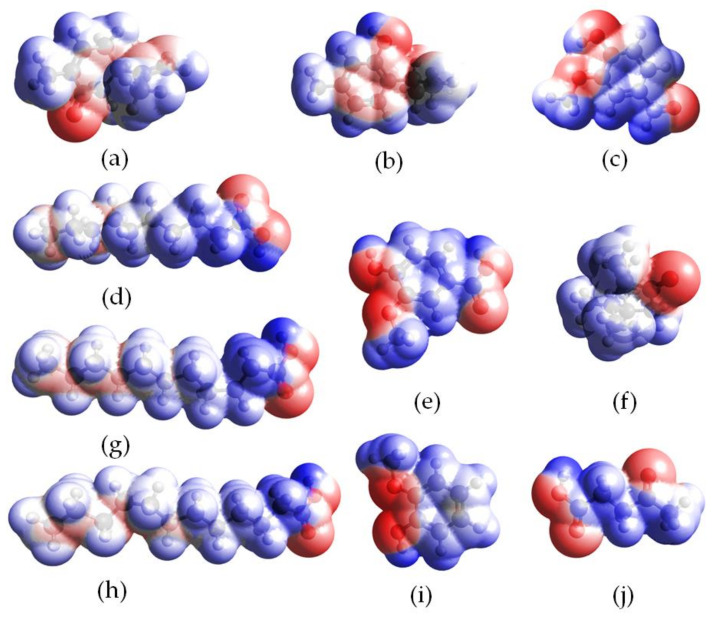
ESP mapped onto total electron density with an isovalue of 0.001 for DES components, i.e., (**a**) carvone, (**b**) thymol, (**c**) vanillin, (**d**) decanoic acid, (**e**) vanillic acid, (**f**) camphor, (**g**) undecanoic acid, (**h**) dodecanoic acid, (**i**) guaiacol and (**j**) levulinic acid. Blue color represents positive charges, red color represents negative charges, and white color represents neutral charges.

**Figure 5:**
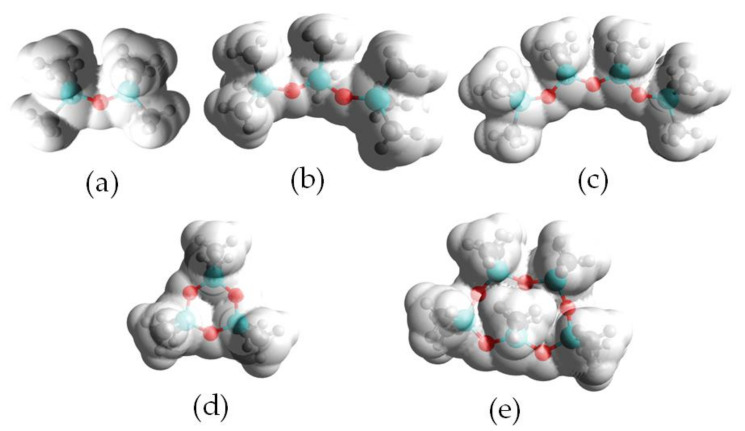
ESP mapped onto total electron density with an isovalue of 0.001 for siloxanes, i.e., (**a**) L2, (**b**) L3, (**c**) L4, (**d**) D3 and (**e**) D5. Blue color represents positive charges, red color represents negative charges, and white color represents neutral charges.

To confirm the results obtained using the σ-profiles, electrostatic potential (ESP) analysis was performed for DES components and selected siloxanes. The electrostatic potential of DESs and siloxanes are mapped onto electron densities in Figure 4 and Figure 5. The positive potential regions are depicted as blue, red indicates negative potential, and white represents the potentials that are close to zero. The obtained results indicated that the electropositive area in DES component structures was located around the hydrogen atoms in the –C-H and –O-H groups. The electronegative area was located around the oxygen atom in the –O– and =O groups, and the neutral region was located around carbon atoms. During the formation of DESs, the electropositive area from one of the DES components (HBD) attracted the electronegative region of the second component (HBA). In this way, strong hydrogen bonds were formed between the DES compounds, resulting in the formation of stable DES. Figure 5 shows that, in all siloxane compounds, only a neutral region could be identified. This indicated that other weaker non-bonded interactions must play the dominant role in the capture of siloxane by DESs.

### 2.2. Synthesis of New DESs 

From a technological point of view, only DESs, which ensure high solubility levels of linear and cyclic siloxanes, should be considered in further research. Therefore, in the next step, only DESs that were characterized by lower activity coefficient values were synthesized, including C:DA, C:UA, and C:DDA, at a 1:1 molar ratio. However, only C:DA and C:UA were in a liquid form at room temperature. Therefore, C:DDA was also synthesized at 2:1 and 3:1 molar ratios, and only C:DDA at a 3:1 ratio was liquid at room temperature. 

### 2.3. An Experimental DESs Structural Characterization

#### 2.3.1. Fourier Transform Infrared Spectroscopy (FT-IR) 

**Figure 6:**
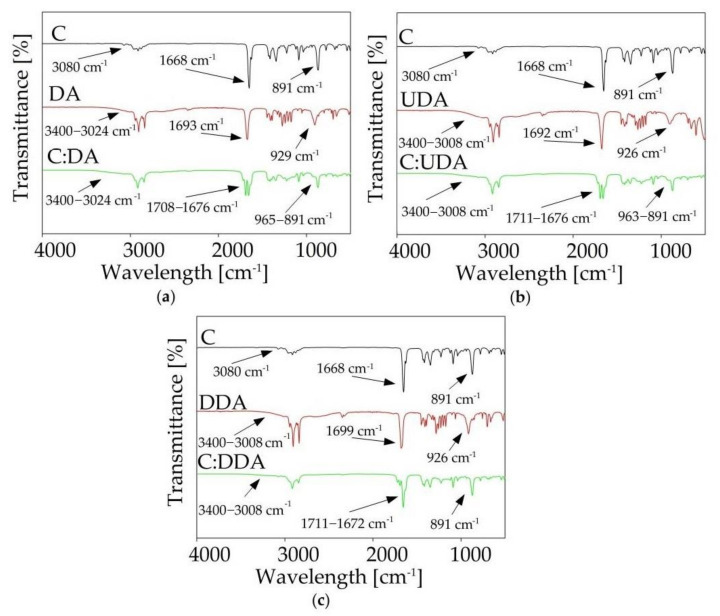
FT-IR spectra of pure components (HBA and HBD) and synthesized DES: (**a**) C:DA (1:1), (**b**) C:UDA (1:1), (**c**) C:DDA (3:1).

FT-IR analysis provided a lot of important information about the structure of the synthesized DES. Based on the peak shifts, it was possible to determine which groups of individual substances were involved in the formation of DES, as well as to determine whether by-products were formed during the synthesis process. Figure 6 shows the spectra of pure substrates (HBA and HBD) and synthesized DESs. In the C:DA (1:1) spectrum, shifts of the stretching carbonyl band vibrations towards higher values, in comparison to both pure HBA (from 1693 cm^−1^ to 1708 cm^−1^) and HBD (from 1668 cm^−1^ to 1676 cm^−1^), were observed. This indicates that both groups, –COOH from DA and =O from C, were involved in the formation of a hydrogen bond. In addition, the γO-H deformation band (929 cm^−1^), which could be observed in the DA spectrum, underwent distortion in the DES spectrum. In the DES spectrum, wide peaks within the wavelength number range from 965 to 891 cm^−1^ were observed. The deformation of the band of the -OH group confirmed the fact that it was involved in the formation of hydrogen bonds. Similar shifts were also observed in the spectra of the remaining DESs (C:UDA (1:1), and *C:*DDA (3:1)). 

#### 2.3.2. Nuclear Magnetic Resonance Spectroscopy (NMR)

**Figure 7:**
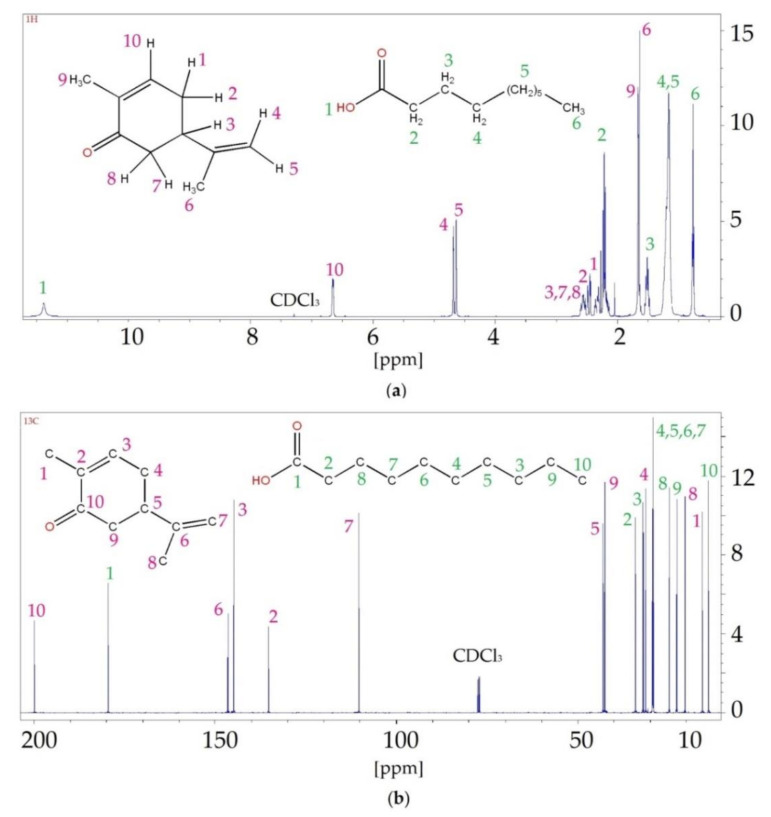
^1^H NMR (**a**) and ^13^C NMR (**b**) spectrum of the C:DA (1:1).

The results of the ^1^H NMR analysis confirmed the formation of hydrogen bonds between HBA (carvone) and various HBDs (i.e., DA, DDA and UDA). Spectra are presented in Figure 7 and Figures S1 and S2. The detailed values of chemical shifts are presented in Table 1, Tables S1 and S2. In the ^1^H NMR spectra, shifts of hydrogen (H1) from the hydroxyl group that was present in the structure of carboxylic acids, towards lower values, were observed for DA (from 11.87 to 11.38 ppm) and DDA (from 11.87 to 10.69 ppm). For UDA, the shift towards higher values, from 10.4 to 11.43 ppm, was observed. The greatest differences in shifts could be observed for DES with DDA (about 0.2 ppm), and the lowest for DES with DA (about 0.1 ppm). This indicated that in DES composed of carvone and DDA, the strongest H-bonding occurred. All changes in the hydrogen signal from HBA were shifted towards lower values, except for H8. Hydrogen H8, which was shifted towards higher values in the DES spectrum, was located close to the oxygen atom from the carbonyl group. This may indicate that hydrogen bonds were formed in the vicinity of H8. 

In this study, ^13^C NMR analysis was also performed. The observed shifts likewise indicated the formation of hydrogen bonds between the carvone and the fatty acids. This was confirmed by the C1 shifts in the carboxyl group in the acids and C10 in the carbonyl group in the carvone. In addition, shifts of carbons (C2 and C9) that were directly connected to the hydroxyl and carbonyl groups were also visible.

In both the ^1^H NMR and ^13^C NMR spectra of all DESs, only signals assigned to the substrates were observed. This indicated that no by-products were formed during the synthesis.

#### 2.3.3. Physicochemical Properties of DESs

The melting point was one of the more important parameters that indicated the formation of DES. The chemical compounds that were used for the DESs synthesis were solid at room temperature. The MP of pure substrates was 25.2, 31.6, 28.6 and 43.2 °C for carvone, decanoic acid, undecanoic acid, and dodecanoic, respectively. For new DESs, a large depression in MP was observed. The melting points of new DESs were −19, −11.7 and −18.8 °C for C:DA (1:1), C:UDA (1:1) and C:DDA (3:1), respectively. 

DES density and viscosity are physicochemical parameters that have a key influence on mass transfer processes [28]. Therefore, the dynamic viscosity of absorbents should be as low as possible, so that DES can be used for absorption processes. These are also parameters that largely depend on the HBD and HBA that are used for the DESs’ synthesis. 

**Figure 8:**
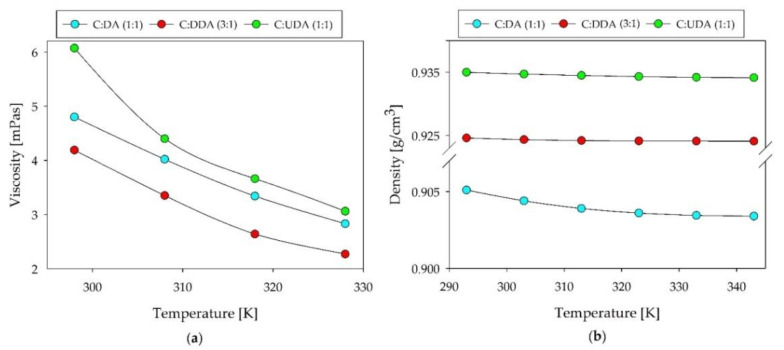
(**a**) Viscosity and (**b**) density of the new DESs.

The obtained results indicated that all of the studied DESs were characterized by relatively low viscosity. At 25 °C, the viscosity of the DESs ranged from 4 to 6 mPas. Compared to other DESs, i.e., tetraethylammonium chloride:oleic acid at a 1:3 molar ratio, and tetrabutylammonium bromide:decanoic acid (1:2), for which the viscosity was above 200 mPas, the obtained viscosity values were much lower [37,54]. This is a favorable feature since when DESs are used as absorption solvents, their viscosity should be as low as possible. The lower viscosity of the absorbents facilitates the mass transfer process. This is consistent with Walden’s rule: the lower the viscosity, the greater the diffusion coefficient, and vice versa. The viscosity of all DESs decreased with increasing temperature, which indicated normal liquid behavior (Figure 8a).

The results of the DESs’ density are presented in Figure 8b. The lowest density was obtained for C:DA (1:1), and the highest for C:UDA (1:1). With the increasing of the temperature from 20 to 70 °C, only slight changes in density could be observed. The largest visible differences were noticeable for C:DA (1:1) (about 0.002), and the lowest for C:DDA (3:1). The densities of the studies DES showed lower densities than water, ranging from 0.905 to 0.935 g/cm^3^.

### 2.4. Absorption Process 

Research on the siloxane absorption process from the model biogas stream was conducted for all synthesized DESs (C:DA (1:1), C:UDA (1:1) and C:DDA (3:1)). In the first part of the studies, as a model biogas, mixtures of nitrogen and siloxanes, such as L2, L3, L4, D3, and D5 at 30 g/m^3^ concentration levels, were used. Pure nitrogen was used to exclude the effect of the matrix on the absorption capacity results. The flow rate was kept constant (50 mL/min) throughout the process. The research was carried out in accordance with the procedure described in previous works [36,39,55,56]. 

**Figure 9:**
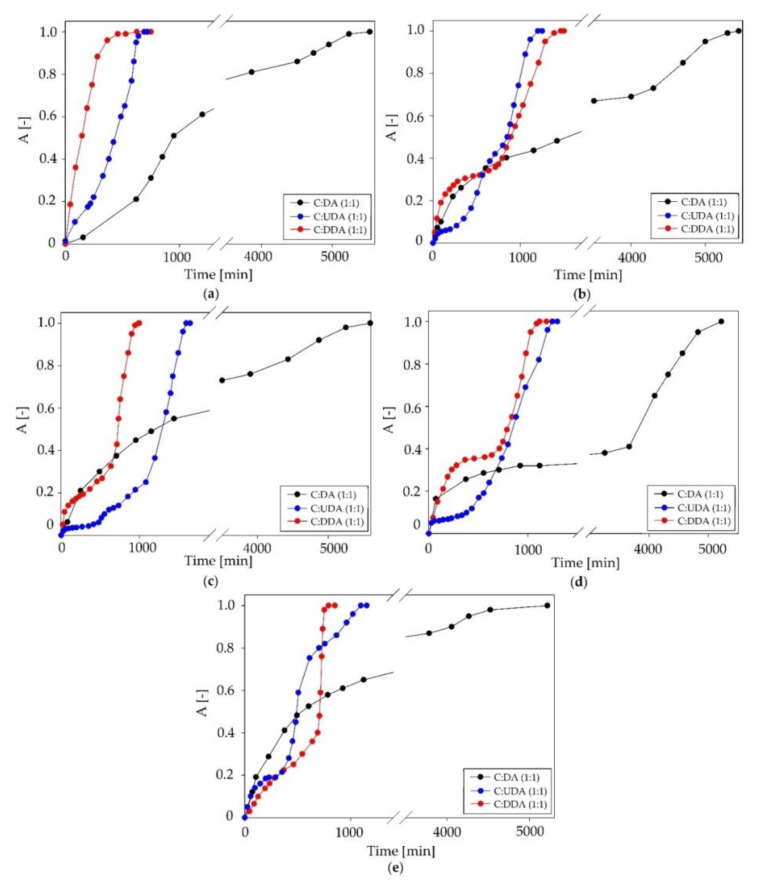
Absorption curves of the studied DESs for individual siloxanes: (**a**) L2, (**b**) L3, (**c**) L4, (**d**) D3 and (**e**) D5.

In this part of the study, the DESs that were characterized by the highest siloxane absorption capacity were selected. The obtained results are presented in Figure 9. All new DESs showed high siloxane absorption capacity. However, the most effective absorbent was DESs composed of C:DA (1:1). C:DA (1:1) displayed effective absorption capacity up to 5600 min. After this time, DES saturated and the process became ineffective. The saturation of DES (C:DA) was fastest for cyclic siloxanes D3 and D4. The time of the effective absorption process was 5217 min. The other DESs (C:UDA (1:1) and C:DDA (3:1)) showed a much shorter effective absorption capacity for siloxanes, as measured in terms of time (1500 and 1650 min). In addition, both DESs (C:UDA and C:DDA) were saturated most rapidly by the volatile linear siloxane (L2) (750 and 720 min), which indicates that the others siloxanes were more easily absorbed than L2. The vapor pressure and polarity of L2 were much greater compared to other linear siloxanes, which may have influenced the reduction in L2 removal from biogas. The cyclic siloxanes had a greater number of Si−O bonds relative to the linear siloxanes, which had great potential to form stronger bonds between siloxanes and DES. This may explain the greater affinity of cyclic siloxanes to-wards DESs.

**Figure 10:**
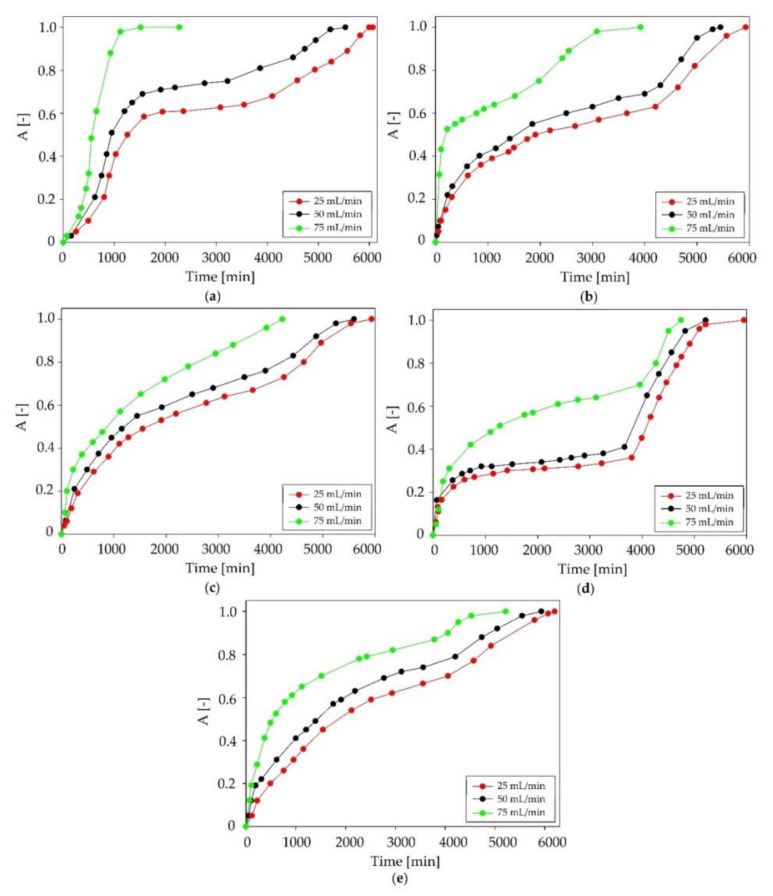
Absorption curves of C:DA (1:1) at various biogas flow rates in the range of 25–75 mL/min for individual siloxanes: (**a**) L2, (**b**) L3, (**c**) L4, (**d**) D3 and (**e**) D5.

In the next part of the study, the most effective DES was subjected to further processes, in which the biogas stream consisted mainly of methane and carbon dioxide at a 2:1 *v*/*v* volume ratio. The concentration of siloxanes remained at the same level (30 g/m^3^). The used concentrations of substances corresponded to the composition of the real biogas streams [13]. For C:DA (1:1), studies on the effect of the biogas flow rate, in the range from 25 to 75 mL/min, on the siloxanes removal efficiency were performed. This range corresponded to the laminar flow rate. The obtained results are presented in Figure 10. The shortest effective siloxane absorption time was obtained for the highest biogas flow of 75 mL/min. In turn, the longest absorption time was obtained at the flow rate of 25 mL/min. This was due to the fact that, with the increasing of the biogas flow rate, the contact time of siloxanes with DES was reduced, which adversely affected the absorption process [36,55]. DES was saturated with the L2, L3 and L4 compounds after about 5500 min (flow rate: 50 mL/min) and 6000 min (flow rate: 25 mL/min), with D3 after about 5000 min (flow rate: 50 mL/min) and 6000 min (25 mL/min), and D5 for both flow rates at about 6000 min. The greatest differences in the absorption time between the flows 75, 50, and 25 mL/min were observed for the linear compounds. This confirms that cyclic siloxanes presented more affinity to DES than linear ones.

**Figure 11:**
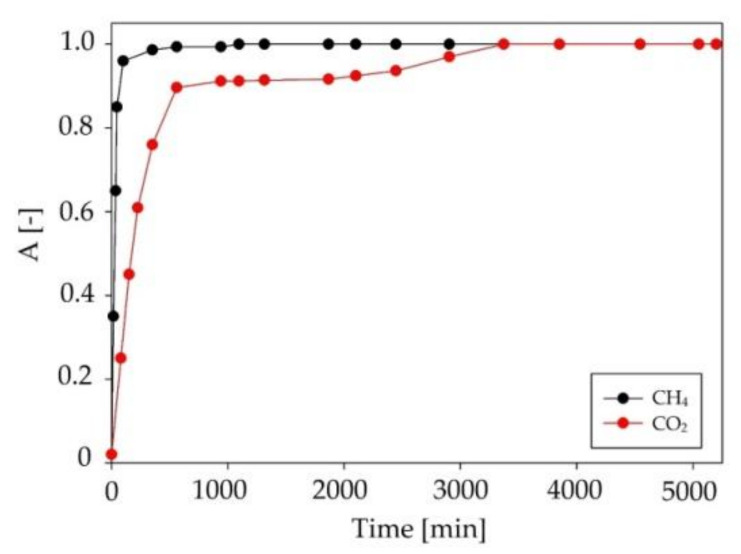
Absorption curves for methane and carbon dioxide in C:DA (1:1).

**Figure 12:**
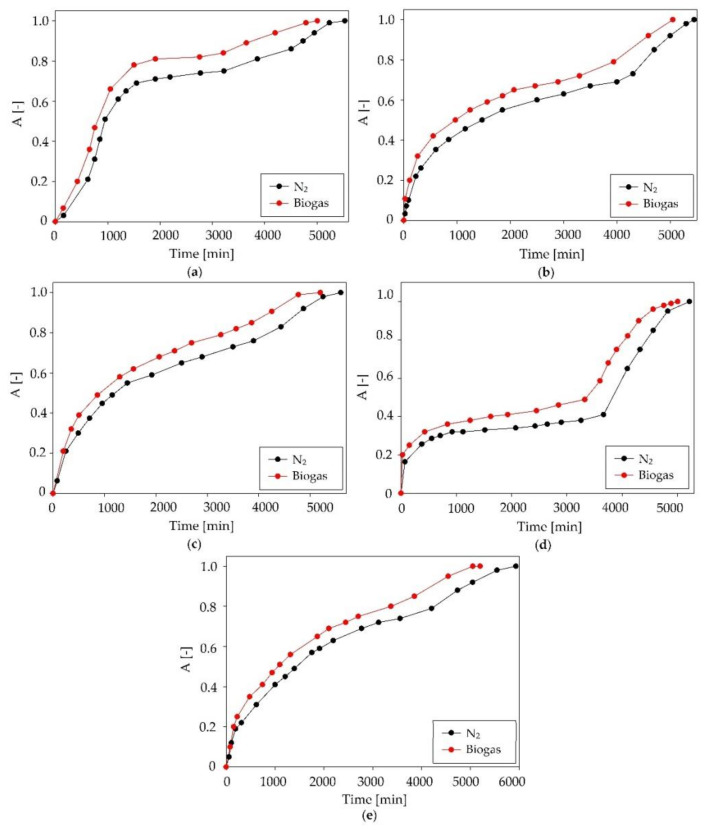
Absorption curves of C:DA (1:1) at various biogas matrix compositions for individual siloxanes: (**a**) L2, (**b**) L3, (**c**) L4, (**d**) D3 and (**e**) D5.

During the absorption process, the content of the main components (CH_4_ and CO_2_) of the model biogas stream was also controlled. The absorption curves for CH_4_ and CO_2_ are presented in Figure 11. The obtained results indicated that, during the initial stage of the absorption process, both the CH_4_ and CO_2_ gases were also absorbed by DES. The C:DA (1:1) became saturated with methane in a very short time (after 50 min). Due to this, the methane loss was about 1%, which was within the acceptable range [55]. In contrast, the process of effectively capturing CO_2_ took much longer, around 500 min. This was due to the presence of active oxygen in the CO_2_ structure that could form competing strong hydrogen bonds with DES. The removal of CO_2_ from biogas is advantageous because it is a gas that substantially reduces the calorific value of biogas [57]. Theoretically, CO_2_ absorption should deteriorate the efficiency of siloxane capture due to the same active groups being present in the pollutant structures. However, due to the low efficiency of CO_2_ removal with DES, only a minor effect of DES saturation with carbon dioxide on the efficiency of siloxane removal could be observed (Figure 12). 

### 2.5. Mechanism of Siloxane Absorption 

#### 2.5.1. FT-IR Analysis

**Figure 13:**
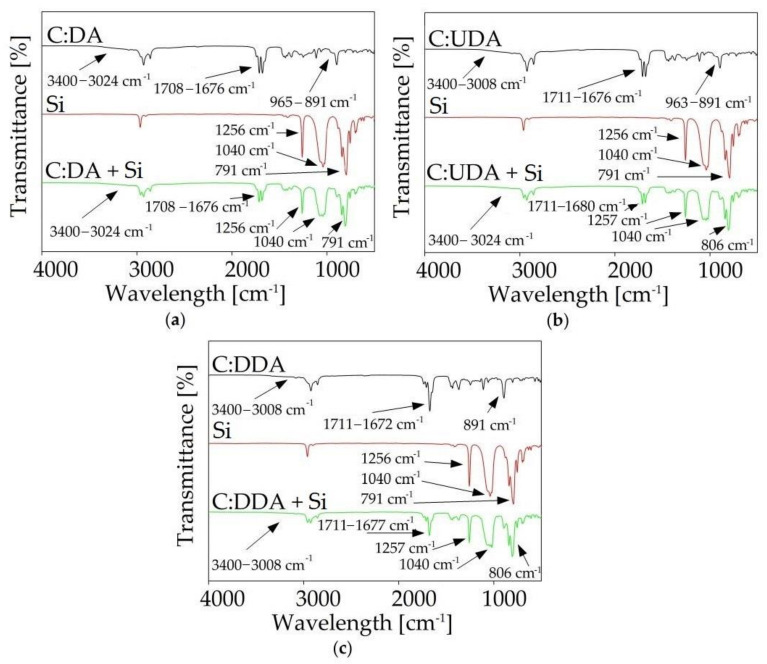
FT-IR spectra before and after siloxanes’ (S) absorption for (**a**) C:DA (1:1), (**b**) C:UDA (1:1) and (**c**) C:DDA (3:1).

To confirm the effectiveness of the biogas purification process, FT-IR analyses of DESs before and after absorption were prepared. The obtained spectra are presented in Figure 13. In the spectra of DES after siloxane absorption, characteristic (Si-O-Si) bands were observed between 780 and 1260 cm^−1^. This confirmed that siloxanes (S) were bound in DESs’ active centers. The lack of band shifts in the vicinity of 3000–3500 cm^−1^ and 1670–1720 cm^−1^ indicated that siloxanes did not combine with DES via hydrogen bonds, and that weaker non-bonded interactions played a major role in the absorption process. 

#### 2.5.2. NMR Analysis

**Figure 14:**
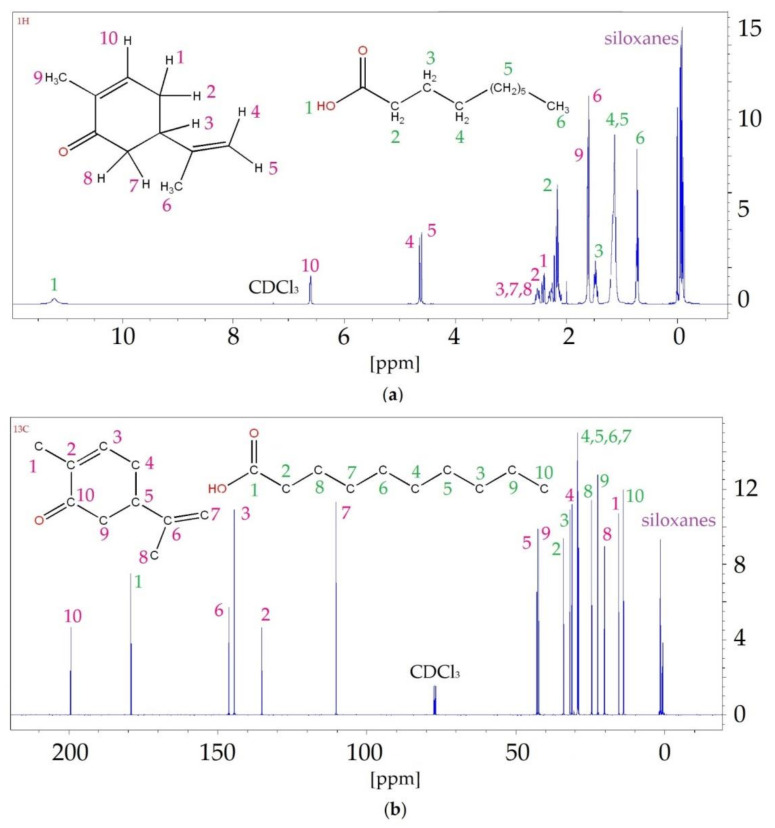
^1^H NMR (**a**) and ^13^C NMR (**b**) spectra for C:DA (1:1) after the absorption process of siloxanes from the model biogas stream.

Additionally, DESs after the absorption process was subjected to ^1^H NMR, and ^13^C NMR analysis (Figure 14, and Figures S3 and S4). The detailed results of shifts values are summarized in Table 2, and Tables S3 and S4. In the ^13^C NMR spectrum, all signals were shifted towards lower values. The greatest differences were visible at the C1 carbon shift (0.3–0.4 ppm) for each acid. Similar shifts were also observed for carvone-derived C10. In both cases, carbon atoms were directly bonded to oxygen atoms. This indicates that both C=O and -COOH groups were involved in the formation of weak non-bonded bonds with siloxanes. The ^1^H NMR spectra showed similar results to those obtained for ^13^C NMR. All signals were shifted towards lower values, and the greatest shift in values was observed for acid H1 (0.16–0.28 ppm). In addition, in both the ^1^H NMR and ^13^C NMR spectra, the signals from the siloxanes were visible. This confirms the effectiveness of the absorption process using DESs.

### 2.6. Desorption Process

**Figure 15:**
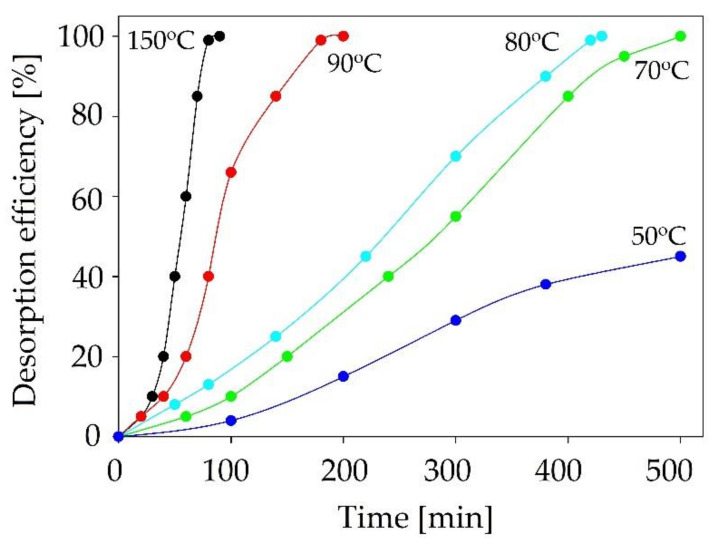
The effect of temperature on C:DA (1:1) in the desorption process.

From an industrial point of view, the absorbent regeneration process is extremely important. The possibility of multiple applications of sorption material significantly reduces the process costs [57]. On the other hand, the regeneration process is considered to be the main disadvantage of the absorption process due to its high energy consumption. Therefore, the desorption step should be optimized. In the studies, the desorption process was performed using the pure nitrogen bubbling process at elevated temperatures in range of 50–150 °C. The absorbent regeneration process was performed for the best absorbent, C:DA (1:1) (Figure 15). The obtained results indicated that the highest desorption efficiency was observed for the process at a temperature of 150 °C. The complete siloxanes desorption was obtained after 90 min. Along with the decrease in process temperature, the desorption time was extended (up to 500 min at 70 °C). Below 70 °C, the desorption process was ineffective. 

**Figure 16:**
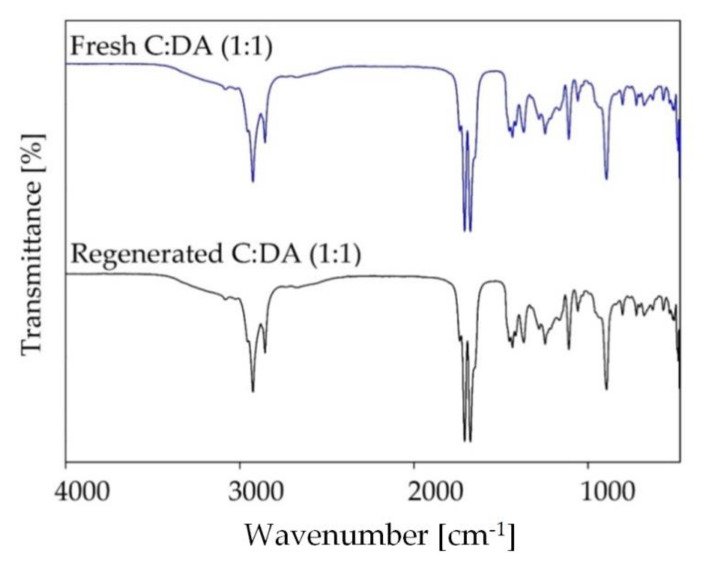
FT-IR spectra of the DES-based absorbent after the regeneration process.

In the further part of the study, FT-IR spectra were obtained for the regenerated DES and compared with the fresh DES spectrum (Figure 16). In the FT-IR spectra, after the desorption process, no characteristic bands, derived from siloxanes, were observed. This indicates that the application of nitrogen bubbling at elevated temperatures is a suitable absorbent regeneration method.

**Figure 17:**
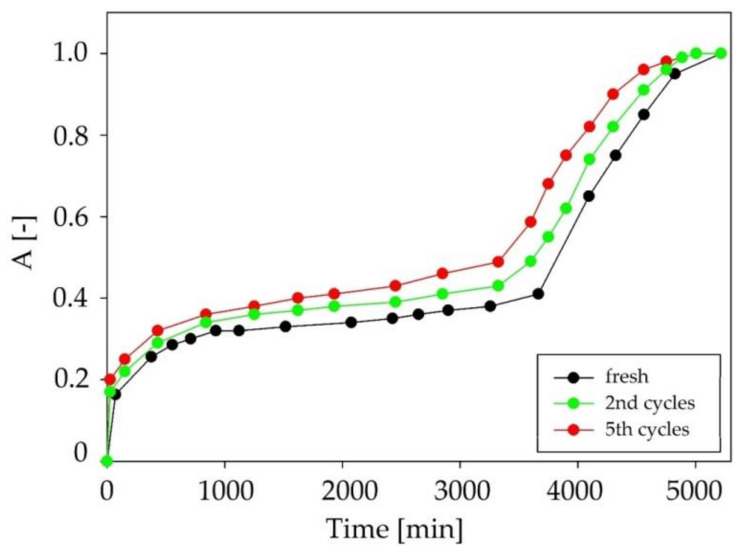
Absorption curves for fresh C:DA (1:1) and after 2 and 5 regeneration cycles.

In addition, C:DA (1:1) showed the ability to complete five regeneration cycles without changing the efficiency of the absorption process. The absorption curves for successive absorption cycles are presented in Figure 17. In comparison with the data in the literature, the obtained results indicated a high efficiency of the regeneration process of DES, without a significant loss of absorption capacity. For example, the adsorption capacity of active carbon decreased by 65% after three siloxane adsorption/desorption cycles at 90 °C [58]. In other studies, a 30% reduction in adsorption capacity was observed after one cycle of the process carried out at a temperature of 160 °C for 4 h [59].

## 3. Materials and Methods

### 3.1. Materials

The following chemicals were used in the studies: carvone (purity: ≥97%), decanoic acid (purity: ≥98%), undecanoic acid (purity: ≥97%), dodecanoic acid (purity: ≥98%), hexamethyldisiloxane (purity: ≥98.5%), octamethyltrisiloxane (purity: ≥98%), decamethyltetrasiloxane (purity: ≥97%), hexamethylcyclotrisiloxane (purity: ≥98%), decamethylcyclopentasiloxane (purity: ≥97%). All standards were purchased from Sigma Aldrich (St. Louis, MO, USA).

The following compressed gases were used to prepare the model biogas stream: nitrogen (purity: N 5.5), methane (purity: N 5.0) and carbon dioxide (purity: N 2.2). These were purchased from Linde Gas (Łódź, Poland).

For the GC analysis, compressed gases, such as helium (purity: N 5.0), nitrogen (purity: N 5.5), air (purity: N 5.0) and hydrogen (purity: N 5), were used. Air was generated by the DK50 compressor with a membrane dryer (Ekkom, Kraków, Poland), while hydrogen was generated by the Precision Hydrogen 1200 Generator (PEAK Scientific, Scotland, UK).

### 3.2. Apparatus

The efficiency of the siloxane absorption process was controlled by gas chromatography (Autosystem XL) (PerkinElmer, Waltham, MA, USA) with a flame ionization detector (FID) (PerkinElmer, Waltham, MA, USA), HP-5 (30 m × 0.25 mm × 0.25 µm) capillary column (Agilent Technologies, Santa Clara, CA, USA), and using TurboChrom 6.1 software (PerkinElmer, Waltham, MA, USA). 

The methane and carbon dioxide contents in the biogas stream were monitored by means of gas chromatography (SRI Instruments, Earl St, Torrance, CA, USA) coupled with a thermal conductivity detector (TCD, SRI Instruments, Earl St, Torrance, CA, USA), and a packed column (Porapak Q 80/100, 2 m × 2 mm) (Restek, Bellefonte, PA, USA). In the investigations, the PeakSimple data system version 4.09 (exclusively licensed by SRI Inc., McLean, VA, USA) was used.

The following apparatuses were used to evaluate the structural and physicochemical properties: a Bruker Tensor 27 spectrometer (Bruker, Billerica, MA, USA) with an ATR adapter and OPUS software (Bruker, Billerica, MA, USA), a Bruker Avance III HD 400 MHz (Bruker, Billerica, MA, USA), a BROOKFIELD LVDV-II + viscometer (Labo-Plus, Warsaw, Poland), a DMA 4500 M (Anton Paar, Graz, Austria), a Bruker Avance III HD 400 MHz (Bruker, Billerica, MA, USA) and a cryostat (HUBER, Edison, NJ, USA).

### 3.3. Procedures

#### 3.3.1. COSMO-RS Studies

The fast screening of 90 DESs composed of natural ingredients, i.e., carvon (C), camphor (Cam), menthol (M), thymol (Th), vanillin (V), guaiacol (Gu), levulinic acid (LA), vanillin acid (VA), decanoic acid (DA), undecanoic acid (UA) and dodecanoic acid (DDA), was prepared using ADF COSMO-RS software (SCM, Netherlands). In the first part of the studies, the geometry optimization of all 90 deep eutectic solvents complexes composed of HBA and HBD in a 1:1 molar ratio was performed using the continuum solvation COSMO model at the BVP86/TZVP theoretical level. The geometry optimization studies were performed in the gas phase in order to find the most stable conformers, and then vibrational analysis was performed to identify the deep eutectic solvent conformer that corresponds to the true energy minimum. Only for the most energetically favorable conformer, full geometry optimization of deep eutectic solvents was prepared. Based on the activity coefficients, the DES with the highest affinity for siloxanes was selected. The activity coefficient was calculated using Equation (1):

(1) $$\ln\left(\gamma_{i}\right)=\frac{\mu_{i}^{DES}-\mu_{i}^{p}}{RT}$$

where: $\mu_{i}^{sol}$—chemical potential of siloxanes in DES;

$\mu_{i}^{p}$—chemical potential of pure siloxanes; 

**R**—universal gas constant (8.314 J/mol);

**T**—temperature (K).

#### 3.3.2. DES Synthesis

**Figure 18:**
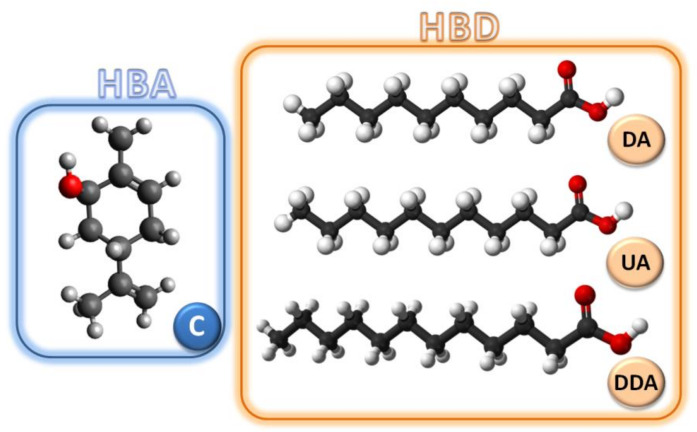
Structures of HBA and HBD used for DES synthesis.

Deep eutectic solvents were synthesized by mixing C with DA, UDA, and DDA at a 1:1 molar ratio. The stirring process was carried out on a magnetic stirrer at 800 RPM, at 60 °C. The obtained liquid DES was allowed to cool to room temperature. The chemical structures of HBA and HB, for the synthesis of DES, are presented in Figure 18.

#### 3.3.3. DES Structural Characterization 

FT-IR spectra were recorded using attenuated total reflection (ATR) with the following operating parameters: spectral range—4000–550 cm^−1^; number of background scans—256; number of sample scans—256; resolution—4 cm^−1^; slit width—0.5 cm. The NMR spectra of the DESs were prepared by weighing 20 mg of a DESs and adding 0.7 mL of chloroform-d1. The measurements were carried out at 20 °C, with the use of the Bruker Avance III HD 400 MHz (Bruker, Billerica, MA, USA).

#### 3.3.4. Physicochemical Properties of DES

The viscosity of the new DES was measured in the temperature range of 25–55 °C at 90 RPM. The density was measured in the temperature range of 20–70 °C. The measurement of uncertainty for the temperature was 0.5 °C. The melting points (MP) were measured using a cryostat. A quantity of 5 mL of the DESs was cooled to −50 °C and then a temperature increase of 1 °C/min was implemented. The MP of DES was determined visually. The point at which the first liquid drop appeared was taken as the melting point.

#### 3.3.5. Preparation of Model Biogas

In the studies, two types of model biogas were used. Both model biogas streams were prepared using the bubbling method. In the first biogas stream, pure N_2_ was passed through a 10-mL vial containing liquid siloxanes mixture (2 mL of L2 and D5, 3 mL of L3 and L4, and 1.6 g of solid D3 siloxane). Gas enriched with siloxanes was diluted with nitrogen in order to reach a 30 g/m^3^ siloxanes concentration in the model biogas stream. In the second biogas stream, nitrogen was replaced with a mixture of CH_4_ and CO_2_ at a 2:1 *v*/*v* volume ratio. 

#### 3.3.6. Absorption Process

**Figure 19:**
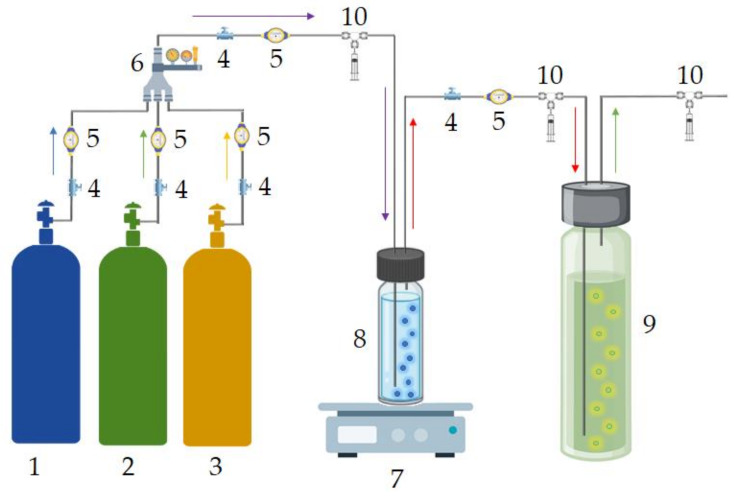
Siloxane absorption installation: 1-N_2_ bottle; 2-CH_4_ bottle; 3-CO_2_ bottle; 4-valve; 5-flowmeter; 6-gas mixer; 7-hotplate; 8-vial with siloxanes; 9-vial with DES; 10-gas sample collection point.

The scheme of the installation for absorption process is shown in Figure 19. In the process, the biogas stream was directed to the absorption column with 50 mL of DES, in which the siloxanes removal process took place based on the bubbling phenomenon. The pure biogas stream was discharged from the top of the absorption column. The absorption process was controlled at the inlet and outlet of the absorption column in terms of the effectiveness of the removal of siloxane impurities. Additionally, the concentration of CH_4_ and CO_2_ during the siloxane absorption process was also examined. 

The absorption efficiency (A) of siloxanes, methane, and carbon was calculated using Equation (2):

(2) $$A=\frac{C_{in}-C_{out}}{C_{in}}$$

where: $C_{in}$—inlet siloxanes, CH_4_, CO_2_ concentration in biogas stream (g/m^3^);

$C_{out}$—outlet siloxanes, CH_4_, CO_2_ concentration in biogas stream (g/m^3^).

After the absorption process, DESs were regenerated by means of pure nitrogen bubbling. The regeneration experiments were carried out at the temperature of 90 °C with a nitrogen flow of 50 mL/min for 2 h. The efficiency of DESs after regeneration process was tested by means of GC analysis. 

#### 3.3.7. Gas Chromatographic Analysis

The siloxanes removal efficiency from the biogas stream was determined using the gas chromatography method coupled with a flame ionization detector (GC-FID). The following chromatographic condition was used: oven temperature program at 40 °C, ramped at 10 °C/min to 200 °C; FID detector temperature of 250 °C; injection port temperature of 200 °C; injection mode split of 5:1; nitrogen as carrier gas (180 mL/min).

The chromatographic methodology was validated based on the linearity, limits of detection (LOD), quantification (LOQ), precision, accuracy, and global uncertainty. Linearity was determined by the direct injection of siloxane standard mixtures at concentrations ranging from 0.005 to 50 g/m^3^. The linearity of calibration was estimated using the correlation coefficient. The LOD was calculated from LOD=3·S/N, where S is analyte signal (mV), and N is noise level (mV). The LOQ was calculated from LOQ=3·LOD. The following results were obtained: LOD in range of 0.004–0.007 g/m^3^, LOQ in range of 0.012–0.021 g/m^3^, R^2^ > 0.997; RSD < 5%.

The composition of the main components of the model biogas stream (methane and carbon dioxide) was monitored by means of gas chromatography coupled with a thermal conductivity detector (GC-TCD). The oven temperature was 40 °C, the detector temperature was 80 °C, the flow rate of the carrier gas (He) was 5 mL/min, and 200 μL of the gaseous sample was introduced into the column. Calibration was performed in the range of 5–50% *v*/*v* and 30–70% for CO_2_ and CH_4_, respectively. The method validation was performed as described above. The following results were obtained: LOD in range of 1–5% *v*/*v*; LOQ in the range of 3–15% *v*/*v*; R^2^ > 0.981; RSD < 6%.

### 4. Conclusions

The paper presents new deep eutectic solvents composed of natural ingredients, including monoterpenes, fatty acids, and polyphenols, as suitable absorbent materials for the capture of siloxanes from biogas. The specific conclusions are as follows:

- Based on the COSMO-RS studies, among 90 eutectic mixtures, DESs composed of carveon and carboxylic acids, i.e., decanoic, undecanoic, and dodecanoic acid, have the highest affinity for siloxanes.
- Hydrogen bonds play a dominant role in the formation of DESs. On the other hand, weaker non-bonded interactions are responsible for the efficiency of the removal of siloxanes from biogas.
- From the industrial point of view, new DESs are characterized by favorable physicochemical properties, i.e., low viscosity and density, and a low melting point, which enable efficient mass transfer even at low temperatures.
- DESs can be easily regenerated by bubbling at elevated temperatures, and their efficiency only slightly decreases after five cycles.
- The proposed absorption procedure based on DESs has great potential for the treatment of real biogas streams due to the high capture of siloxanes’ selectivity.

## Figures and Tables

**Table 1:** Chemical shift values from the ^1^H NMR and ^13^C NMR spectra for C:DA (1:1).

^1^H NMR							
HBA		DES		HBD		DES	
atom	δ (ppm)	atom	δ (ppm)	atom	δ (ppm)	atom	δ (ppm)
H1	2.45	H1	2.31	H1	11.87	H1	11.38
H2	2.35	H2	2.30	H2	2.21	H2	2.21
H3	2.69	H3	2.56	H3	1.54	H3	1.51
H4	4.76	H4	4.68	H4	1.33	H4	1.19
H5	4.81	H5	4.64	H5	1.26	H5	1.15
H6	1.76	H6	1.63	H6	0.88	H6	0.76
H7	2.57	H7	2.48				
H8	2.35	H8	2.44				
H9	1.78	H9	1.66				
H10	6.77	H10	6.65				
**^13^C NMR**							
C1	15.63	C1	15.48	C1	180.81	C1	179.50
C2	135.45	C2	135.27	C2	34.26	C2	33.96
C3	144.40	C3	144.74	C3	31.99	C3	31.77
C4	31.30	C4	31.12	C4	29.53	C4	29.32
C5	42.55	C5	42.89	C5	29.38	C5	29.17
C6	146.69	C6	146.40	C6	29.38	C6	29.16
C7	110.47	C7	110.33	C7	29.19	C7	28.98
C8	20.50	C8	20.26	C8	24.79	C8	24.62
C9	43.20	C9	42.35	C9	22.77	C9	22.55
C10	199.34	C10	199.7	C10	14.13	C10	13.92

**Table 2:** Chemical shift values from ^1^H NMR and ^13^C NMR spectra from individual DES components before and after the siloxane absorption process.

**^1^H NMR**							
**C**		**C:DA (1:1)+Si**		**DA**		**C:DA (1:1)+Si**	
atom	δ (ppm)	atom	δ (ppm)	atom	δ (ppm)	atom	δ (ppm)
H1	2.31	H1	2.26	H1	11.38	H1	11.22
H2	2.35	H2	2.31	H2	2.21	H2	2.17
H3	2.56	H3	2.52	H3	1.51	H3	1.48
H4	4.68	H4	4.64	H4	1.19	H4	1.16
H5	4.64	H5	4.61	H5	1.15	H5	1.13
H6	1.63	H6	1.59	H6	0.76	H6	0.74
H7	2.48	H7	2.44				
H8	2.44	H8	2.40				
H9	1.66	H9	1.62				
H10	6.65	H10	6.60				
**^13^C NMR**							
**C**		**C:DA (1:1)+Si**		**DA**		**C:DA (1:1)+Si**	
C1	15.48	C1	15.38	C1	179.50	C1	179.20
C2	135.27	C2	135.30	C2	33.96	C2	33.83
C3	144.74	C3	144.40	C3	31.77	C3	31.72
C4	31.12	C4	31.08	C4	29.32	C4	29.28
C5	42.89	C5	42.83	C5	29.17	C5	29.14
C6	146.40	C6	146.10	C6	29.16	C6	29.14
C7	110.33	C7	110.10	C7	28.98	C7	28.94
C8	20.26	C8	20.17	C8	24.62	C8	24.55
C9	42.35	C9	42.32	C9	22.55	C9	22.49
C10	199.7	C10	199.40	C10	13.92	C10	13.83

## Data Availability

Not applicable.

## Supplementary Materials

The following are available online at https://www.mdpi.com/article/10.3390/ijms22179551/s1. Figure S1: ^1^H NMR (a) and ^13^C NMR (b) spectra of C:DDA (3:1); Figure S2: ^1^H NMR (a) and ^13^C NMR (b) spectra of C:UDA (1:1); Figure S3: ^1^H NMR (a) and ^13^C NMR (b) spectra for C:DDA (3:1) after the absorption process; Figure S4: ^1^H NMR (a) and ^13^C NMR (b) spectra for C:UDA (1:1) after the absorption process; Table S1: Chemical shift values from the ^1^H NMR and ^13^C NMR spectra for C:DDA (3:1); Table S2: Chemical shift values from the ^1^H NMR and ^13^C NMR spectra for C:UDA (1:1). Table S3: Chemical shift values from ^1^H NMR and ^13^C NMR spectra of C:DDA (3:1) after the absorption process. Table S4: Chemical shift values from ^1^H NMR and ^13^C NMR spectra of C:UDA (1:1) after the absorption process.
