# Supplementary material for: New Carvone-Based Deep Eutectic Solvents for Siloxanes Capture from Biogas
Source: Int J Mol Sci. 2021 Sep 2;22(17):9551. doi: 10.3390/ijms22179551 (PMC8431123; doi:10.3390/ijms22179551)
Supplement: Supplementary file 1 [file ijms-22-09551-s001.zip › ijms-1347551-supplementary.pdf]

Electronic Supplementary Information (ESI)

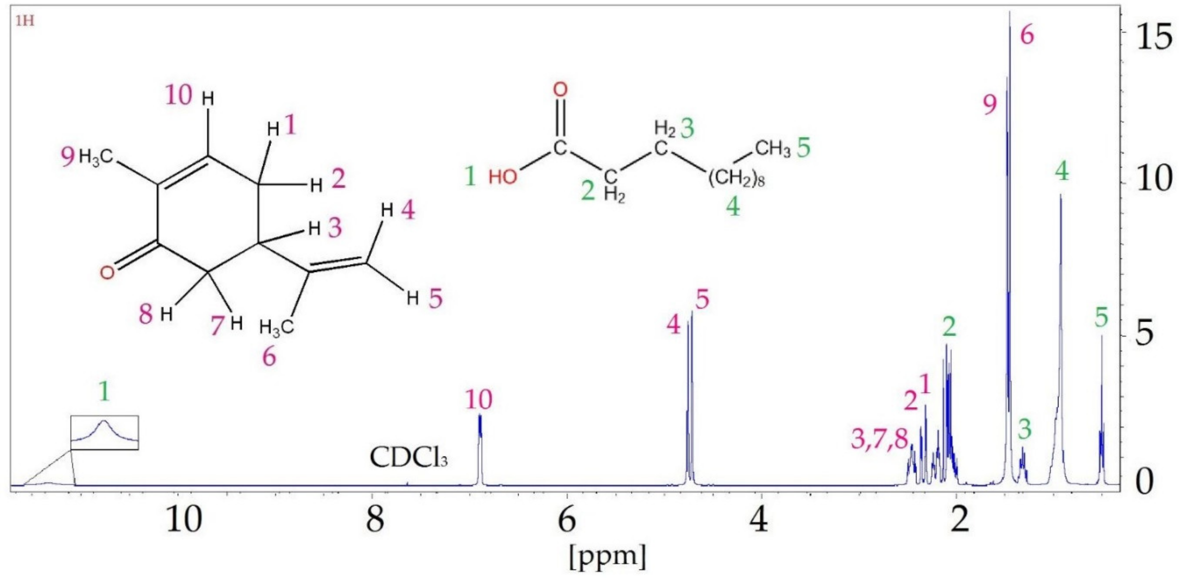

(**a**)

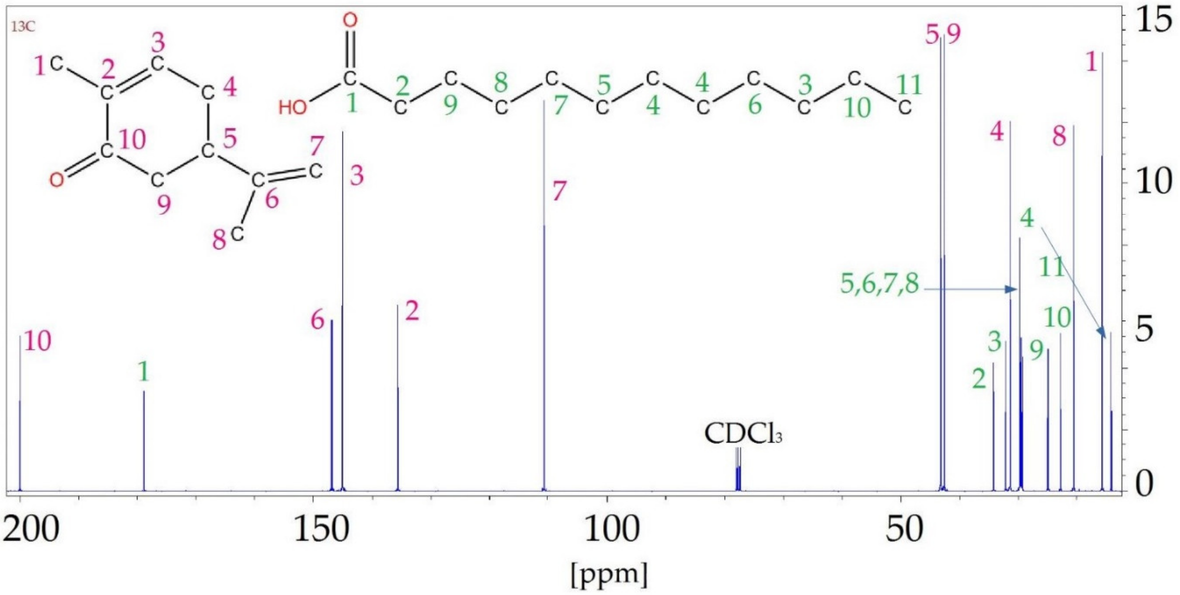

(**b**)

Figure S1. ^{1}H-NMR (a) and ^{13}C-NMR (b) spectra of C:DDA (3:1).

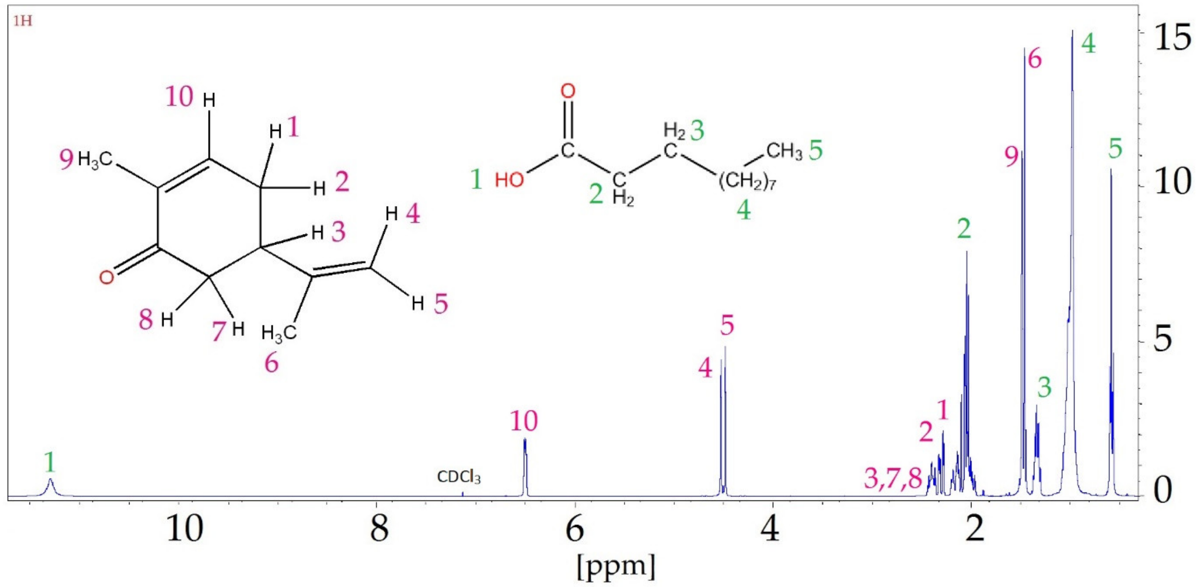

(**a**)

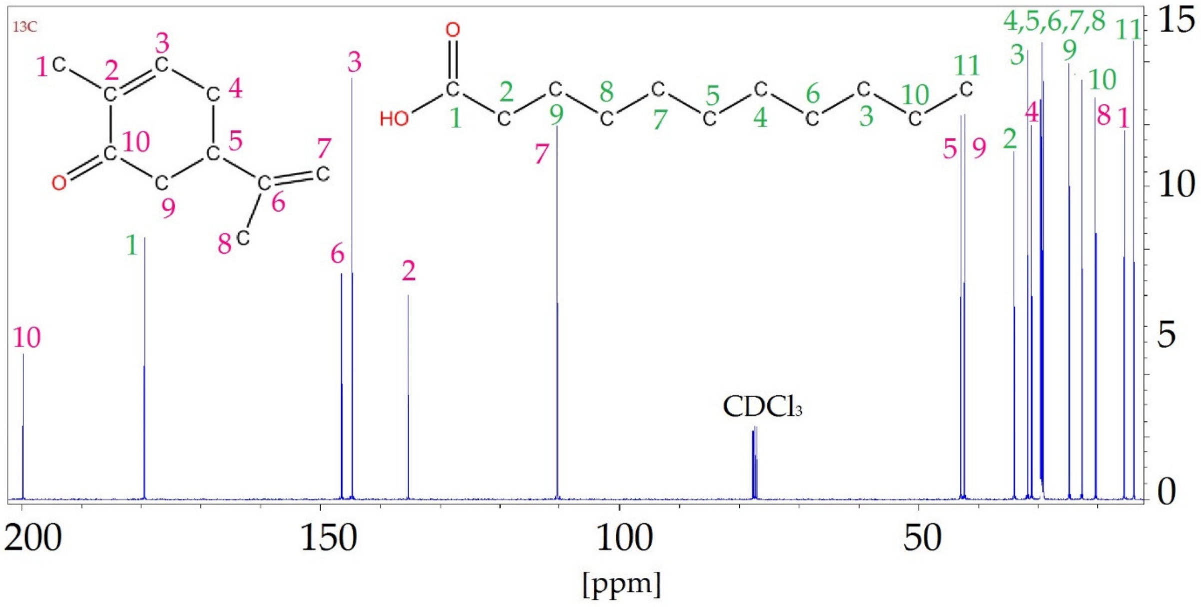

(**b**)

Figure S2. ^{1}H NMR (a) and ^{13}C NMR (b) spectra of C:UDA (1:1).

Table S1. Chemical shift values from the ^1H NMR and ^{13}C NMR spectra for C:DDA (3:1).

^1H NMR							
**C**		**C:DDA (3:1)**		**DDA**		**C:DDA (3:1)**	
atom	δ [ppm]	atom	δ [ppm]	atom	δ [ppm]	atom	δ [ppm]
H1	2.45	H1	2.26	H1	11.87	H1	10,69
H2	2.30	H2	2,30	H2	2,34	H2	2,16
H3	2.69	H3	2.51	H3	1.64	H3	1.46
H4	4.76	H4	4.63	H4	1.26	H4	1.1
H5	4.81	H5	4.59	H5	0.88	H5	0.71
H6	1.76	H6	1.58				
H7	2.57	H7	2.42				
H8	2.35	H8	2.38				
H9	1.78	H9	1.60				
H10	6.77	H10	6.60				
**^{13}C NMR**							
**C**		**C:DDA (3:1)**		**DDA**		**C:DDA (3:1)**	
C1	15.63	C1	15.46	C1	180.74	C1	178.39
C2	135.45	C2	135.17	C2	34.21	C2	33.87
C3	144.40	C3	144.62	C3	31.99	C3	31.77
C4	31.30	C4	31.06	C4	29.68	C4	29.48
C5	42.55	C5	42.87	C5	29.52	C5	29.32
C6	146.69	C6	146.43	C6	29.42	C6	29.20
C7	110.47	C7	110.31	C7	29.34	C7	29.15
C8	20.50	C8	20.28	C8	29.15	C8	28.96
C9	43.20	C9	42.30	C9	24.75	C9	24.62
C10	199.34	C10	199.47	C10	22.76	C10	22.52
				C11	14.13	C11	13.93

Table S2. Chemical shift values from the ^1H NMR and ^{13}C NMR spectra for C:UDA (1:1).

^1H NMR							
**C**		**C:UDA (1:1)**		**UDA**		**C:UDA (1:1)**	
atom	δ [ppm]	atom	δ [ppm]	atom	δ [ppm]	atom	δ [ppm]
H1	2.45	H1	2.32	H1	10.4	H1	11.43
H2	2.30	H2	2.36	H2	2.34	H2	2.22
H3	2.69	H3	2.57	H3	1.63	H3	1.52
H4	4.76	H4	4.69	H4	1.27	H4	1.16
H5	4.81	H5	4.65	H5	0.88	H5	0.77
H6	1.76	H6	1.64				
H7	2.57	H7	2.50				
H8	2.35	H8	2.46				
H9	1.78	H9	1.67				
H10	6.77	H10	6.65				
**^{13}C NMR**							
**C**		**C:UDA (1:1)**		**UDA**		**C:UDA (1:1)**	
C1	15.63	C1	15.48	C1	180.76	C1	179.51
C2	135.45	C2	135.29	C2	34.25	C2	33.95
C3	144.40	C3	144.76	C3	32.00	C3	31.80
C4	31.30	C4	31.12	C4	29.66	C4	29.47

C5	42.55	C5	42.89	C5	29.54	C5	29.36
C6	146.69	C6	146.46	C6	29.41	C6	29.22
C7	110.47	C7	110.37	C7	29.37	C7	29.17
C8	20.50	C8	20.29	C8	29.18	C8	28.98
C9	43.20	C9	42.34	C9	24.77	C9	24.61
C10	199.34	C10	199.74	C10	22.77	C10	22.56
				C11	14.14	C11	13.94

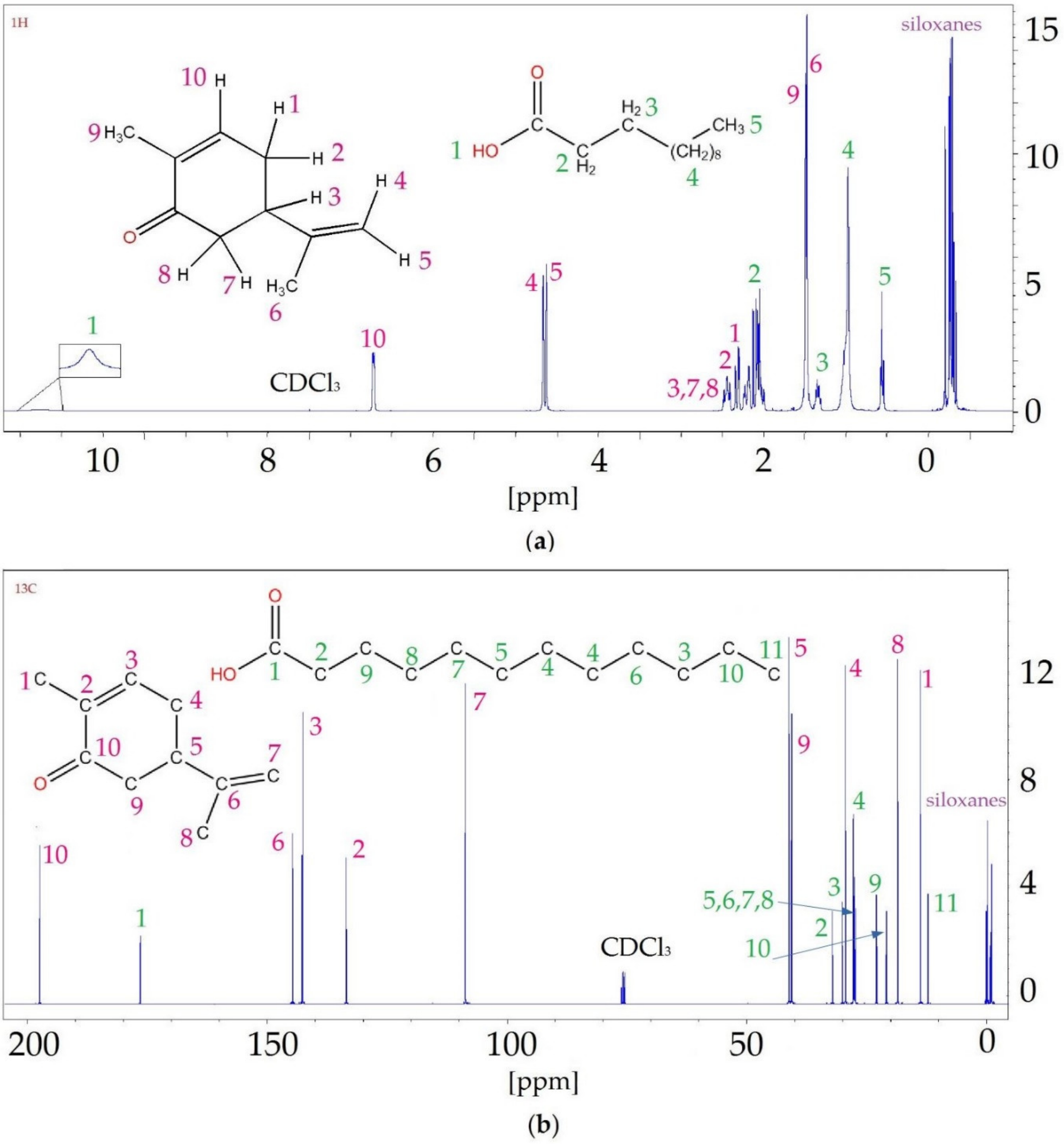

Figure S3. ^{1}H NMR (a) and ^{13}C NMR (b) spectra for C:DDA (3:1) after the absorption process.

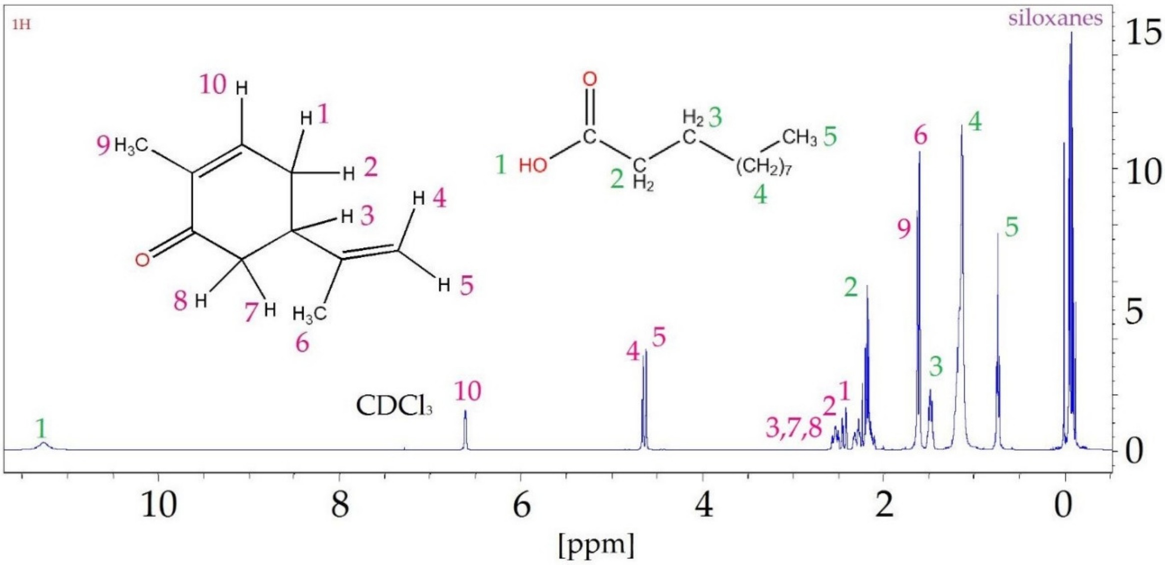

(a)

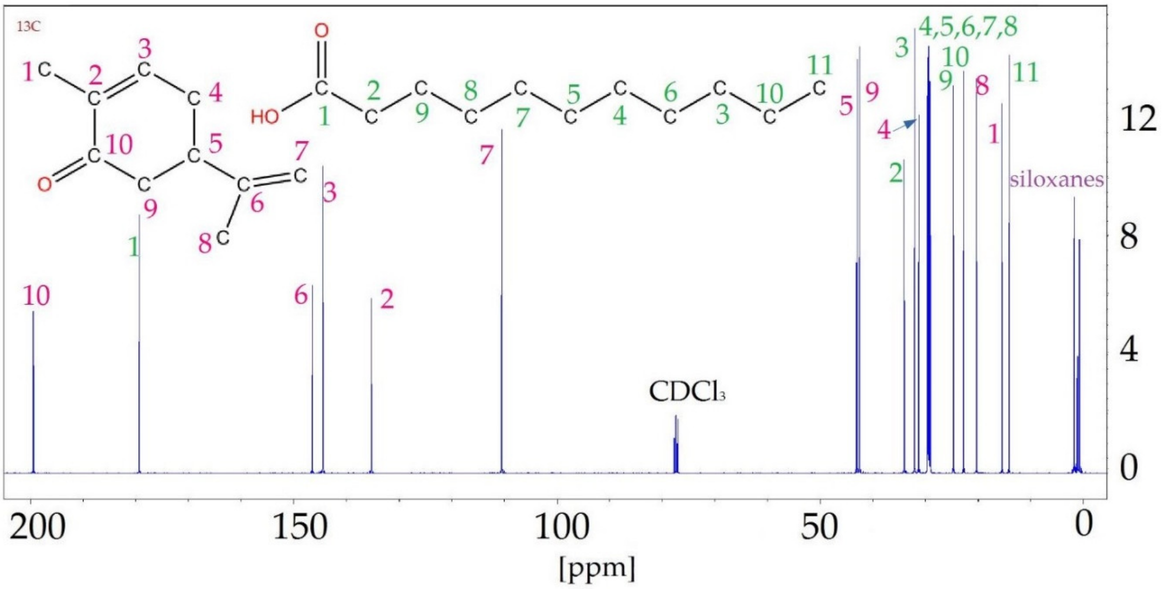

(b)

Figure S4. ^{1}H NMR (a) and ^{13}C NMR (b) spectra for C:UDA (1:1) after the absorption process.

Table S3. Chemical shift values from ^{1}H NMR and ^{13}C NMR spectra of C:DDA (3:1) after the absorption process.

^{1}H NMR							
**C**		**C:DDA (1:1)+Si**		**DDA**		**C:DDA (1:1)+Si**	
atom	δ [ppm]	atom	δ [ppm]	atom	δ [ppm]	atom	δ [ppm]
H1	2.26	H1	2.21	H1	10.69	H1	10.41
H2	2.30	H2	2.25	H2	2.16	H2	2.11
H3	2.51	H3	2.46	H3	1.46	H3	1.41
H4	4.63	H4	4.58	H4	1.10	H4	1.06
H5	4.59	H5	4.54	H5	0.71	H5	0.67
H6	1.58	H6	1.54				
H7	2.42	H7	2.36				
H8	2.38	H8	2.32				
H9	1.60	H9	1.55				
H10	6.60	H10	6.55				
**^{13}C NMR**							
**C**		**C:DDA (1:1)+Si**		**DDA**		**C:DDA (1:1)+Si**	
C1	15.46	C1	15.35	C1	178.39	C1	178.02
C2	135.17	C2	135.13	C2	33.87	C2	33.77
C3	144.62	C3	144.25	C3	31.77	C3	31.72
C4	31.06	C4	31.00	C4	29.48	C4	29.45
C5	42.87	C5	42.78	C5	29.32	C5	29.29
C6	146.43	C6	146.35	C6	29.20	C6	29.16
C7	110.31	C7	110.26	C7	29.15	C7	29.11
C8	20.28	C8	20.15	C8	28.96	C8	28.91
C9	42.30	C9	42.26	C9	24.62	C9	24.56
C10	199.47	C10	199.04	C10	22.52	C10	22.46
				C11	13.93	C11	13.84

Table S4. Chemical shift values from ^{1}H NMR and ^{13}C NMR spectra of C:UDA (1:1) after the absorption process.

^{1}H NMR							
**C**		**C:UDA (1:1)+Si**		**UDA**		**C:UDA (1:1)+Si**	
atom	δ [ppm]	atom	δ [ppm]	atom	δ [ppm]	atom	δ [ppm]
H1	2.32	H1	2.27	H1	11.43	H1	11.26
H2	2.36	H2	2.32	H2	2.22	H2	2.18
H3	2.57	H3	2.53	H3	1.52	H3	1.49
H4	4.69	H4	4.58	H4	1.16	H4	1.14
H5	4.65	H5	4.54	H5	0.77	H5	0.74
H6	1.64	H6	1.54				
H7	2.50	H7	2.36				
H8	2.46	H8	2.32				
H9	1.67	H9	1.55				
H10	6.65	H10	6.55				
**^{13}C NMR**							
**C**		**C:UDA (1:1)+Si**		**UDA**		**C:UDA (1:1)+Si**	
C1	15.48	C1	15.39	C1	179.51	C1	179.19
C2	135.29	C2	135.21	C2	33.95	C2	33.86
C3	144.76	C3	144.41	C3	31.80	C3	31.77
C4	31.12	C4	31.09	C4	29.47	C4	29.44
C5	42.89	C5	42.83	C5	29.36	C5	29.34
C6	146.46	C6	146.31	C6	29.22	C6	29.14
C7	110.37	C7	110.28	C7	29.17	C7	29.15
C8	20.29	C8	20.18	C8	28.98	C8	28.95
C9	42.34	C9	42.36	C9	24.61	C9	24.58
C10	199.74	C10	199.26	C10	22.56	C10	22.53
				C11	13.94	C11	13.88
